# Divergent mitochondrial responses and metabolic signal pathways secure the azole resistance in Crabtree-positive and negative *Candida* species

**DOI:** 10.1128/spectrum.04042-23

**Published:** 2024-03-05

**Authors:** Meng Zhou, Jingwen Peng, Kun Ren, Yu Yu, Dongmei Li, Xiaodong She, Weida Liu

**Affiliations:** 1 Department of Medical Mycology, Institute of Dermatology, Chinese Academy of Medical Sciences and Peking Union Medical College, Nanjing, China; 2 Jiangsu Key Laboratory of Molecular Biology for Skin Diseases and STIs, Nanjing, China; 3 Department of Critical Care Medicine, Nanjing Jinling Hospital, Affiliated Hospital of Medicine School, Nanjing University, Nanjing, China; 4 Centers for pharmaceutical preparations, Institute of Dermatology, Chinese Academy of Medical Sciences and Peking Union Medical College, Nanjing, China; 5 Department of Microbiology & Immunology, Georgetown University Medical Center, Washington, DC, USA; 6 Center for Global Health, School of Public Health, Nanjing Medical University, Nanjing, China; Agroscope, Nyon, Switzerland

**Keywords:** azoles resistance, mitochondria, proteome, *Candida *species

## Abstract

Azole drugs are the main therapeutic drugs for invasive fungal infections. However, azole-resistant strains appear repeatedly in the environment, posing a major threat to human health. Several reports have shown that mitochondria are associated with the virulence of pathogenic fungi. However, there are few studies on the mechanisms of mitochondria-mediated azoles resistance. Here, we first performed mitochondrial proteomic analysis on multiple *Candida* species (*Candida albicans*, *Nakaseomyces glabrata*, *Pichia kudriavzevii,* and *Candida auris*) and analyzed the differentially expressed mitochondrial proteins (DEMPs) between azole-sensitive and azole-resistant *Candida* species. Subsequently, we performed Kyoto Encyclopedia of Genes and Genomes (KEGG) analysis, gene ontology analysis, and protein-protein interaction network analysis of DEMPs. Our results showed that a total of 417, 165, and 25 DEMPs were identified in resistant *C. albicans*, *N. glabrata,* and *C. auris*, respectively. These DEMPs were enriched in ribosomal biogenesis at cytosol and mitochondria, tricarboxylic acid cycle, glycolysis, transporters, ergosterol, and cell wall mannan biosynthesis. The high activations of these cellular activities, found in *C. albicans* and *C. auris* (at low scale), were mostly opposite to those observed in two fermenter species—*N. glabrata* and *P. kudriavzevii*. Several transcription factors including Rtg3 were highly produced in resistant *C. albicans* that experienced a complex I activation of mitochondrial electron transport chain (ETC). The reduction of mitochondrial-related activities and complex IV/V of ETC in *N. glabrata* and *P. kudriavzevii* was companying with the reduced proteins of Tor1, Hog1, and Snf1/Snf4.

**IMPORTANCE:**

*Candida* spp. are common organisms that cause a variety of invasive diseases. However, *Candida* spp. are resistant to azoles, which hinders antifungal therapy. Exploring the drug-resistance mechanism of pathogenic *Candida* spp. will help improve the prevention and control strategy and discover new targets. Mitochondria, as an important organelle in eukaryotic cells, are closely related to a variety of cellular activities. However, the role of mitochondrial proteins in mediating azole resistance in *Candida* spp. has not been elucidated. Here, we analyzed the mitochondrial proteins and signaling pathways that mediate azole resistance in *Candida* spp. to provide ideas and references for solving the problem of azole resistance. Our work may offer new insights into the connection between mitochondria and azoles resistance in pathogenic fungi and highlight the potential clinical value of mitochondrial proteins in the treatment of invasive fungal infections.

## INTRODUCTION

Fungi are widely distributed in nature and yet are closely associated with human daily life. Pathogenic fungi adversely affect human health, causing billions of infections and millions of deaths annually worldwide (1). At present, the majority of clinical isolates from invasive fungal infection (IFI) are mostly classified as *Candida* spp., *Cryptococcus neoformans*, and *Aspergillus* spp (2).

Like other eukaryotic microorganisms and humans, fungal cells have membrane-bound nuclei and organelles and share many common metabolic processes with humans, which leaves us a less potentialities to develop new antifungal agents (3). Currently, only four classes of antifungal agents are used in clinic: azoles, polyenes, echinocandins, and the base analog fluorocytosine (4). Among them, azoles are the primary drugs for the treatment and prevention of fungal infections due to their broad antibacterial spectrum, low toxicity, and high efficiency. However, with the wide and increased usage of azoles, the problem of fungal resistance tends to be prominent, resulting in a serious challenge for antifungal treatment (5). To date, the prevalence of azoles resistance and newly reported echinocandin-resistant *Candida* become a great concern, as drugs in both classes are recommended as first line therapy for patients with invasive fungal infections. Therefore, identification of the new drug targets could be a resolution to aid a rational use of currently used antifungal applications (6–8), which, thus, requires us to deepen understanding of the resistance mechanisms with antifungals such as azoles.

Mitochondria are known to serve as the powerhouses of the eukaryotic cells since it contributes to the most portion of adenosine triphosphate (ATP) generation in the cells (9). Beyond being the energy source, the cumulative evidence has pointed that mitochondria regulate a continuum of cellular functions, spanning from physiological metabolism to stress responses and death in fungal cells, thereby influencing the host responses. A number of studies have demonstrated that the mitochondrial oxidative phosphorylation (OXPHOS) in *Candida albicans* is crucial for virulence and hyphal formation (10, 11), biofilm development (12), cell wall biosynthesis (13), and the stress adaptation via regulating different signal transduction pathways (14–16). The failure of these functions reduces the innate immune cell responses and cytokine production (13, 17) but increases the susceptibility of fungi to drug therapy (18). Oxidative phosphorylation is the process in mitochondria where energy released from substance oxidation is utilized to synthesize ATP via the electron transport chain (complexes I, II, III, IV, and so forth). Impaired mitochondrial OXPHOS function, specifically in mitochondrial complex I (CI), of *C. albicans* mutants rendered them highly susceptible to azoles (18). However, the impact of azoles resistance and adaptation to drug-induced stress on fungal OXPHOS function has not been clearly elucidated (19). And, additional studies have provided compelling evidence that damage or mutation in fungal oxidative phosphorylation can lead to a decrease in fungal sensitivity or confer resistance to targeted drugs, primarily by inducing remodeling of the fungal cell wall. For instance, mutants of Fzo1, responsible for mitochondrial fusion in *C. albicans*, exhibit an increased susceptibility to azole drugs in addition to a defective mitochondrial morphology (20, 21).

Expression activation of drug efflux pumps (CDR1, CDR2, and MDR1) is one of the important mechanisms to confer the fungal resistance to azoles, which have also been shown in *Candida* strains with mutation in ETC subunits, mitochondrial structural proteins, and enzymes (18). In our previous study, the fluconazole susceptibility of *C. albicans* is increased by the combination of ETC complex inhibitors, especially when CI inhibitors piericidin A and C12E8 were used (18). Moreover, in *Nakaseomyces glabrata* (previously named *C. glabrata*), decreased azoles susceptibility was linked to mitochondrial deficiency and the upregulation of ATP binding cassette transporter *CgCDR1* or *CgCDR2* (22). However, the mechanisms governing the regulation of specific mitochondrial functions and respiration in response to azoles stress remain largely unknown. Here, the primary objective of our study was to elucidate the contribution of mitochondria to *Candida* resistance by conducting a comprehensive analysis of mitochondrial proteomics in azole-resistant and susceptible *Candida* strains, which is helpful to identify novel drug targets and devise combination regimens to extend the efficacy of azoles.

## MATERIALS AND METHODS

### Fungal strains

Strains included in this study (Table 1) include four *Candida* species. The azole-resistant strain for each paired sample set was defined as resistant to at least 2–3 azoles in the reference studies. All the strains included in this experiment were obtained from the Fungal Center of the Medical Microbial (Toxic) Species Collection Center of the Ministry of Health, China. Prior to use in this study, all experimental strains were frozen in liquid nitrogen. The thawed strain was streaked in YPD agar plates at 30°C for 48 h for the following studies. A single yeast colony was selected and cultured in YPD liquid medium at 30°C and shaking at 200 rpm/min for 4–6 h for exponential growth or growth condition otherwise indicated.

**TABLE 1:** Paired strains for each *Candida* species and fluconazole MICs for proteomic analysis

Species	Strains	MIC_90_
*C. albicans*	C103 (ATCC90028)	2
	Ca1052	64
*C. glabrata* (*Nakaseomyces glabrata*）	Y104	16
	Y10b (ATCC2001)	64
*C. auris*	10913 (CBS12768)	2
	12768 (CBS12768)	256
*C. krusei* (*Pichia kudriavzevii*）	C6a	64

### Blue native polyacrylamide gel electrophoresis

Prior to mitochondria preparation, the overnight cell cultures of wild-type *C. albicans* (SC5314) in YPD were collected and suspended into the fresh YPD medium in the absence or the presence of 2, 8, and 32 µg/mL fluconazole and incubated at 30°C, 200 rpm for 4 h. Mitochondrial protein preparation followed the mitochondria isolation was purposely to reduce other protein complex contamination (19). Twenty microliters of each sample (60 to 80 µg of protein) was loaded onto a Blue native polyacrylamide gel electrophoresis (BN-PAGE) gradient gel (4%–16%) (Invitrogen, Inc.). Electrophoresis was performed in an X-Cell SureLock mini-cell system (Invitrogen) with cathode buffer (50 mM Tricine) and anode buffer containing 150 mM bis-Tris (pH 7.0; or 75 mM imidazole), and 0.02% Serva Blue G-250, supplemented with 0.02% DDM. An in-gel enzyme assay for OXPHOS CI was accomplished by incubating the gel with 0.2 mM NADH–0.2% nitroblue tetrazolium for 1 h. Reactions were stopped by fixing the gels in 45% methanol in 10% (vol/vol) acetic acid, and then, gels were de-stained overnight in the same solution. Image of the band density for each protein complex was analyzed using ImageJ software (1.52a).

### ATP quantification

First, c103, Ca1052, Y104, Y10b, 10913, and 12768 were prepared as suspensions with a density of 5 × 10^3^ CFU/mL. The suspension was added to a 96-well plate as per 100 µL/well. The 96-well plates were placed in incubators and incubated for 24 h at 35°C. ATP levels were then measured with the CellTiter-Glo luminescent cell viability assay (Beyotime) according to the manufacturer’s instructions. Finally, a Synergy H4 microplate reader (BioTek) was used to measure the luminescence signal.

### Mitochondrial protein isolation

Mitochondrial protein extraction was carried out using a Fungal Mitochondrial Protein Extraction Kit (Bebo biology) according to the manufacturer’s instructions. First, the fungal precipitate was collected and suspended with 2 mL of extracting solution A and vortexed and centrifugated at 500 *g* for 5 min. Next, centrifuge the precipitate three times under different centrifugal conditions according to the instructions. Subsequently, the collected precipitate was supplemented with 100 µL of solution B, thoroughly mixed and oscillated at 4°C for 20–30 min, and then centrifuged at 14,000 *g* for 15 min. Finally, the collected supernatant contained the fungal mitochondrial proteins.

### Data-independent acquisition in liquid chromatography coupled to tandem mass spectrometry detection

To verify that proteins could be distained, reduced, alkylated, and digested in trypsin-containing gels, proteins were loaded into an SDS-PAGE gel, and migration was halted just between the stacking and splitting gels. For eluting the peptide, samples were dissolved by 0.1% (vol/vol) formic acid in water and then by 0.1% (vol/vol) formic acid in acetonitrile. After the peptides were dissolved, they were separated using a nano-ACQUITY UPLC M-Class system (USA ultrahigh performance liquid phase system, Waters).

The scanning range of high-accuracy survey scan (MS1) was set to 400–1,800 *m*/*z* with mass size as 60,000 and the scanning resolution as 15,000. The data acquisition mode was obtained by the data-dependent (DDA) program, which selected the parent ions of the top 20 peptide segments with the strongest signal intensity. After the first-order scan, these peptide segments entered the higher-energy collisional dissociation collision cell. The fragmentation energy of 28 eV was used for fragmentation.

The second-order mass spectrometry analysis was also performed in the data acquisition mode by using the DDA program. Mass spectrometry was setting to 3e6 for the automatic gain control, 10,000 ions for the signal threshold. The maximum ion implantation time was set to 50 ms, and the dynamic exclusion time of tandem mass spectrometry was scanned to 45 s in order to prevent repeatedly scanning parent ions.

### Identification of differentially expressed proteins in fungi

For each paired samples, the ratio of the quantification means of two sets of biological replicates was obtained to present the fold change (FC) of each protein. The *P*-value < 0.05 employed in the *t*-test was chosen to identify the significant differences. The |fold change| ≥ 2 and FDR < 0.05 were considered differentially expressed mitochondrial proteins (DEMPs). All the changes on mitochondrial proteins were mapped in the volcano graph by using volcano-mapping tools (https://www.xiantao.love/products).

### GO and KEGG enrichment analysis

GO analysis (http://www.ncbi.nlm.nih.gov/COG/) was used to investigate the functional connection of different mitochondrial proteins, which included biological processes, cell composition, and molecular function. Kyoto Encyclopedia of Genes and Genomes (KEGG) performed a pathway analysis that identified the critical pathways related to the differential expression of mitochondrial proteins. A *P*-value < 0.05 was statistically significant in both bioinformatic analyses.

### PPI network construction

STRING is used to analyze the protein-protein interaction (PPI) network among DEMPs in a free and open-source database (https://string-db.org/).

## RESULTS

### Assembly of mitochondrial OXPHOS protein complex in *C. albicans* was severely reduced in the presence of high concentration of azole

**Fig 1:**
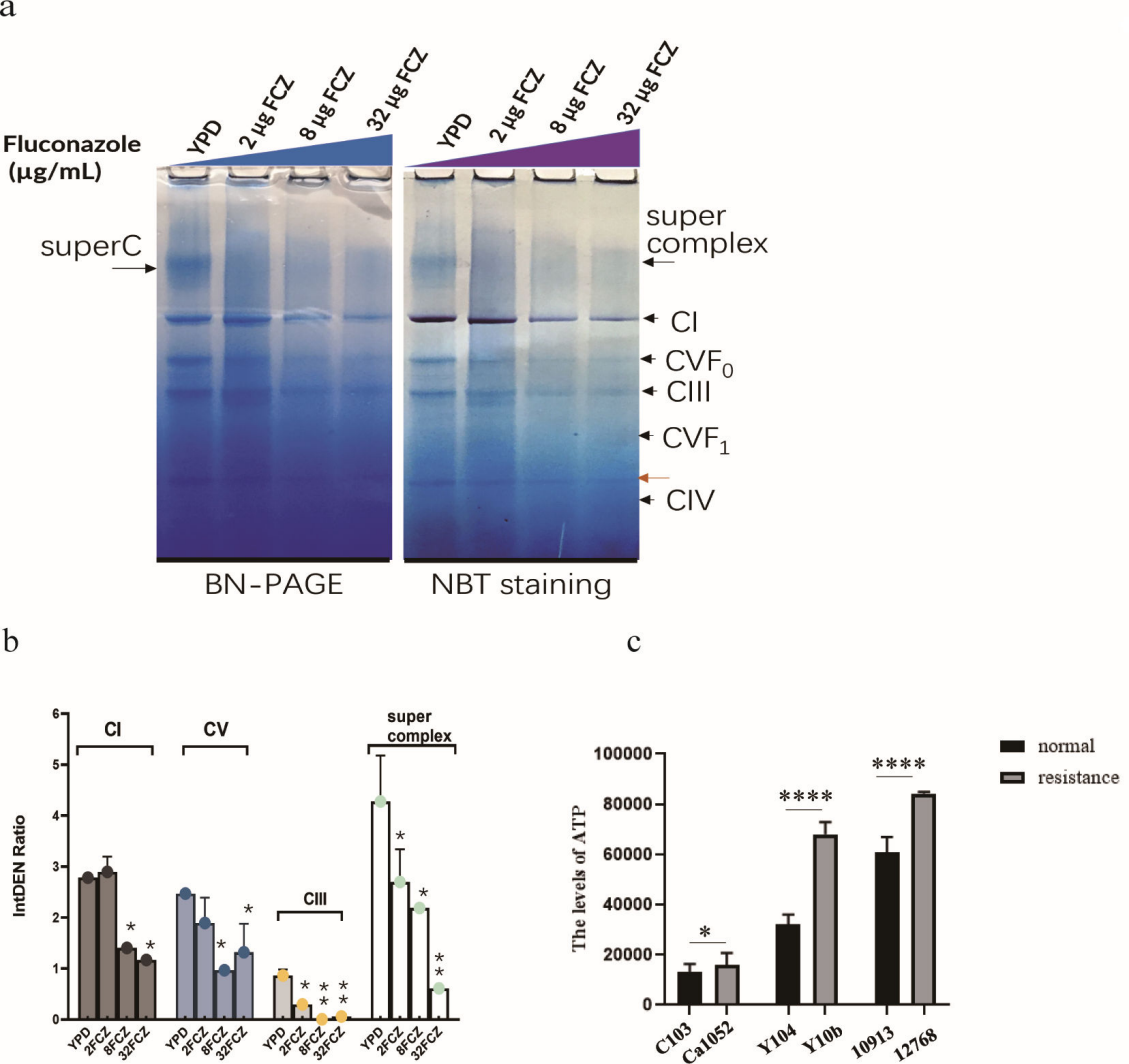
The effects of different concentrations of azole on assembly of OXPHOS protein complex in mitochondria of *Candida albicans*. Each electron transport chain complex was visualized (**a**) and quantified (**b**) by BN-PAGE analysis of indicate condition. (**c**) ATP levels of resistance strains and normal strains (*C. albicans*, *N. glabrata,* and *C. auris*).

To investigate the impact of azoles on fungal mitochondrial OXPHOS, we employed BN-PAGE analysis to explore the effects of fluconazole (FCZ) on the electron transfer chain complexes of OXPHOS in *Candida albicans* (SC5314). As shown in Fig. 1a and b, the effect of 2 µg/mL FCZ did not impact CI and complex V (CV) but resulted in a reduction of complex III (CIII) and supercomplex. FCZ at concentrations of 8 and 32 µg/mL markedly inhibited CI, CV, CIII, and supercomplex. Furthermore, CIII and CV seemed more vulnerable than CI in response to azole stress in the growth environment. Mitochondria are known to produce ATP through OXPHOS (23). ATP levels of resistant and normal strains were determined by ATP detection assay (Fig. 1c), suggesting that the ATP levels of drug-resistant *C. albicans*, *N. glabrata,* and *C. auris* were significantly higher than those of normal *Candida* species.

### Differential expression analysis of mitochondrial proteins in azoles susceptible and -resistant *Candida* species

**Fig 2:**
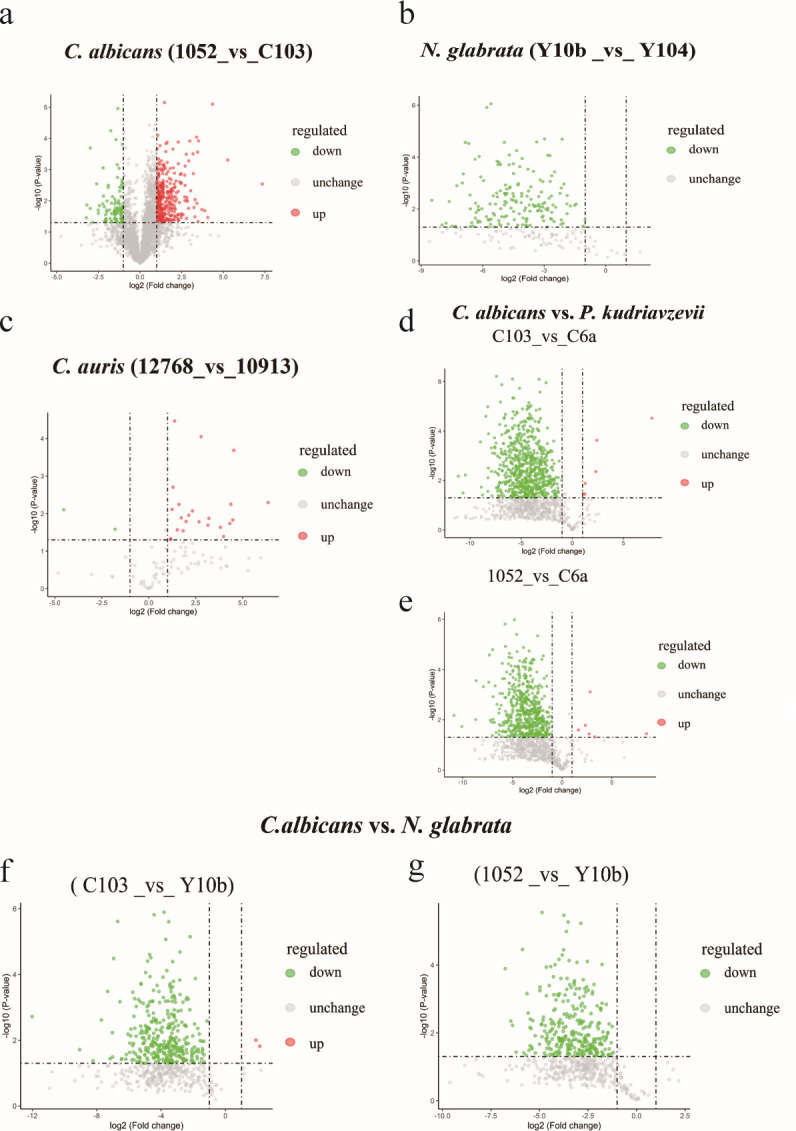
Differential mitochondrial protein expression patterns can be identified by mass spectrometry in *Candida* species. Volcano plot of DEMPs in resistant *C. albicans* compared with normal *C. albicans* (**a**), resistant *N. glabrata* compared with normal *N. glabrata* (**b**), resistant *C. auris* compared with normal *C. auris* (**c**), resistant *C. albicans* compared with *P. kudriavzevii* (d), normal *C. albicans* compared with *P. kudriavzevii* (**e**), resistant *C. albicans* compared with resistant *N. glabrata* (**f**), and normal *C. albicans* compared with resistant *N. glabrata* (**g**) by proteomic analysis based on mass spectrometry.

To further explore the mitochondrial mechanisms responsible for azoles resistance, we employed proteomic analysis to profile the mitochondrial proteins of azoles sensitive and - resistant *Candida* species. As shown in Fig. 2a, a total of 417 DEMPs were identified in paired *C. albicans* strains (1052 vs C103), in which 318 proteins (76.3%) were upregulated and 99 (23.7%) were downregulated (fold change >2.0, *P* < 0.05). While in *N. glabrata* (Y10b vs Y104), a total of 165 mitochondrial DEMPs were discovered of which all (100%) proteins were downregulated (Fig. 2b). Additionally, we found much less mitochondrial DEMPs in *C. auris* (12768 vs 10913), with 23 (92%) proteins upregulated and 2 (8%) proteins downregulated (Fig. 2c).

It is generally known that *P. kudriavzevii* have intrinsic resistance to or poor sensitivity to azoles. Consequently, to further probe the mitochondrial DEMPs that cause intrinsic and acquired resistance to azoles, we performed a comparative analysis between *C. albicans* and *P. kudriavzevii* (C6a) or *N. glabrata*, respectively. Intriguingly, among the obtained DEMPs (C103 or 1052 vs C6a), downregulated proteins accounted for the majority (Fig. 2d and e). Similarly, this massively downregulated DEMPs pattern was also showed in resistant *N. glabrata* vs *C. albicans* strains (C103 or 1052 vs Y10b) (Fig. 2f and g).

### KEGG enrichment pathway analysis and GO analysis of DEMPs in different *Candida* species

**Fig 3:**
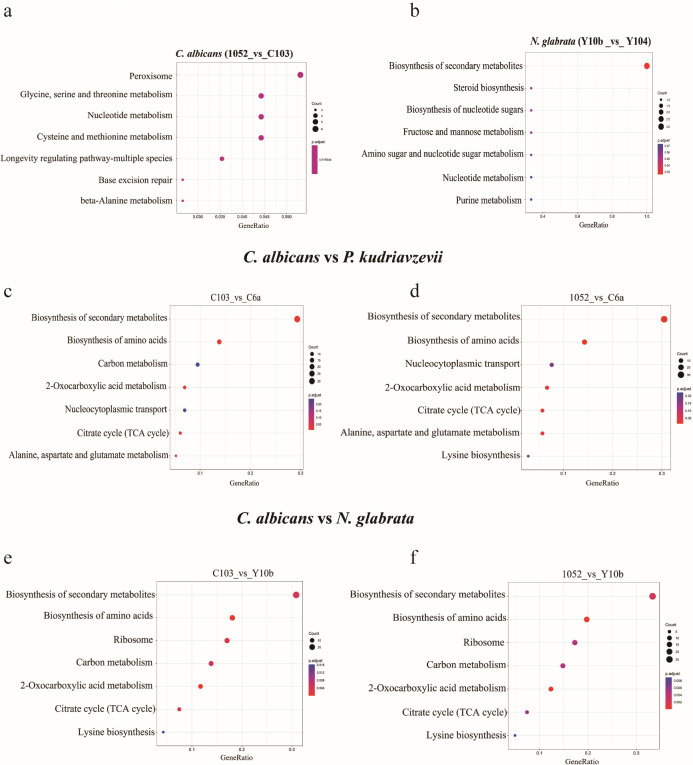
Significant KEGG pathways of DEMPs. KEGG analysis with enriched items by DEMPs in resistant *C. albicans* compared with normal *C. albicans* (**a**), resistant *N. glabrata* compared with normal *N. glabrata* (**b**), resistant *C. albicans* compared with *P. kudriavzevii* (**c**), normal *C. albicans* compared with *P. kudriavzevii* (**d**), resistant *C. albicans* compared with resistant *N. glabrata* (**e**), and normal *C. albicans* compared with resistant *N. glabrata* (**f**).

**Fig 4:**
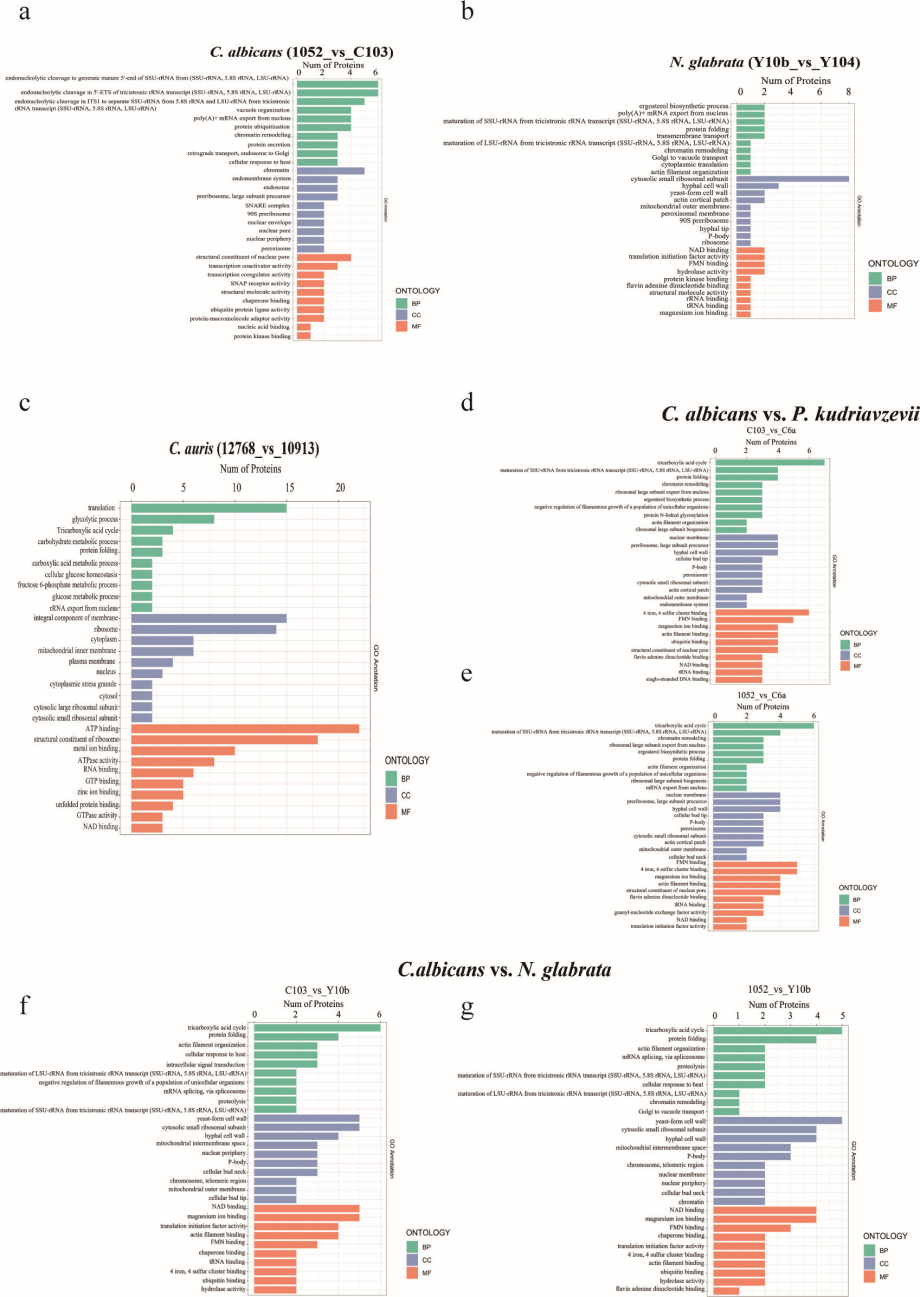
Significant GO terms of DEMPs. GO analysis with enriched items by DEMPs in resistant *C. albicans* compared with normal *C. albicans* (**a**), resistant *N. glabrata* compared with normal *N. glabrata* (**b**), resistant *C. auris* compared with normal *C. auris* (**c**), resistant *C. albicans* compared with *P. kudriavzevii* (**d**), normal *C. albicans* compared with *P. kudriavzevii* (**E**), resistant *C. albicans* compared with resistant *N. glabrata* (**f**),and normal *C. albicans* compared with resistant *N. glabrata* (**g**).

To investigate the signaling pathways and biological functions associated with these DEMPs, KEGG pathway analysis and GO enrichment analysis were performed. In *C. albicans*, they were mainly enriched in peroxisome, amino acid metabolism, and nucleotide metabolism (Fig. 3a). In addition, DEMPs in *C. albicans* were mainly related to rRNA maturation, vacuole organization, protein ubiquitination, and chrome remolding (Fig. 4a). In *N. glabrata*, DEMPs were found in amino acid metabolism, nucleotide sugar metabolism, and mannose/fructose metabolism (Fig. 3b), linked to ribosome maturation, protein folding, and vacuole transporters (Fig. 4b). A notable distinction between the two species was the presence of ergosterol biosynthesis in *N. glabrata*, which is absent in *C. albicans*. In *C. auris*, the commonly enriched GO terms for DEMPs included translation, glycolytic process, tricarboxylic acid cycle (TCA), integral component of membrane, and ATP binding (Fig. 4c).

As shown in Fig. 3c through f, DEMPs (*P. kudriavzevii* vs *C. albicans*) and DEMPs (*N. glabrata* vs *C. albicans*) were equally enriched in the biosynthesis of secondary metabolites, biosynthesis of amino acids, 2-oxocarboxylic acid metabolism, and TCA cycle. Besides, common enriched GO terms for DEMPs (*P. kudriavzevii* vs *C. albicans*) and DEMPs (*N. glabrata* vs *C. albicans*) included TAC, maturation of SSU-rRNA, protein folding, hyphal cell wall, NAD binding, and four iron, four sulfur cluster binding (Fig. 4d through g).

### The protein-protein interaction network and predication of possible regulators

**Fig 5:**
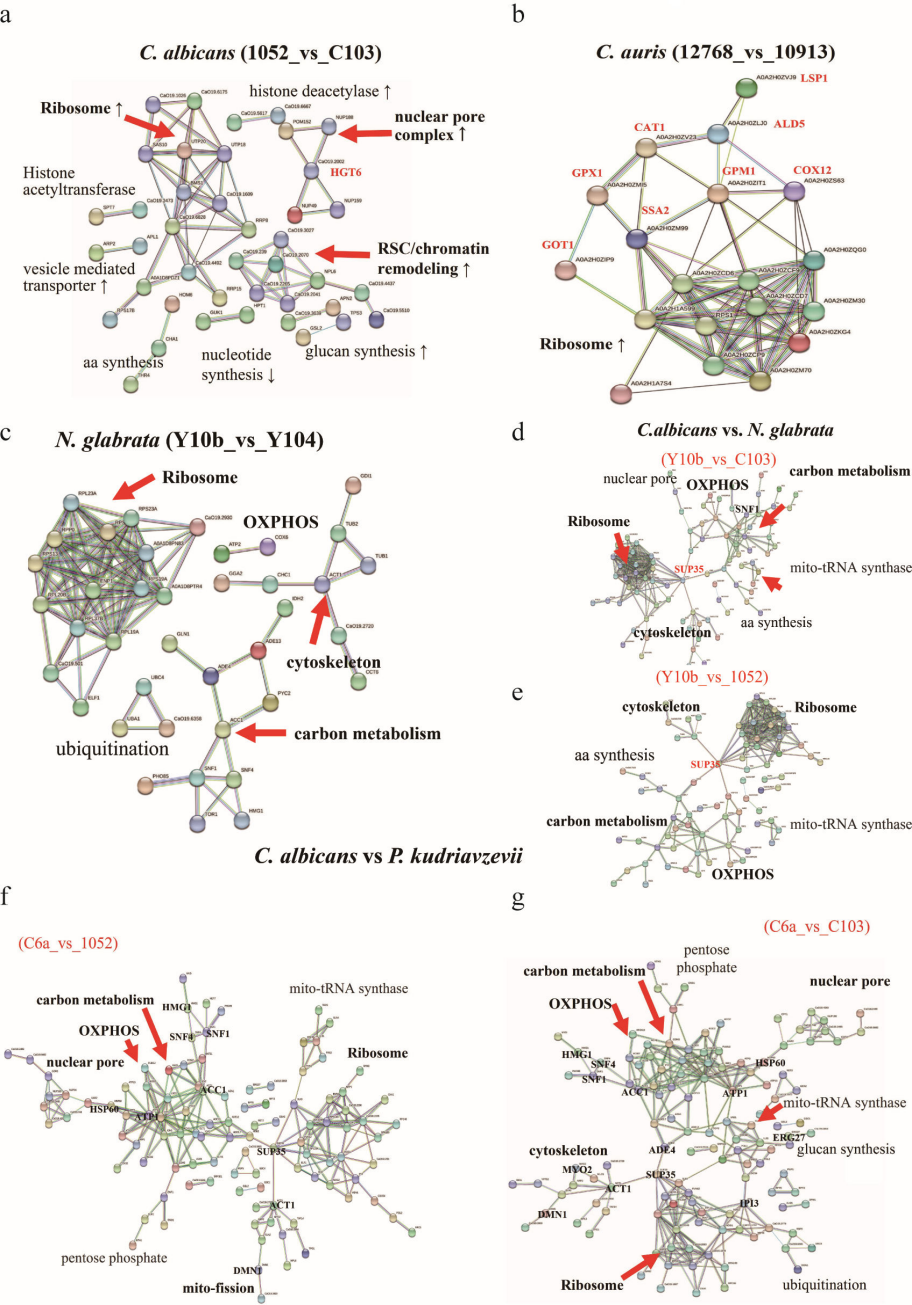
Protein-protein interaction networks designed with STRING. Protein interaction network analysis of DEMPs in resistant *C. albicans* compared with normal *C. albicans* (**a**), resistant *N. glabrata* compared with normal *N. glabrata* (**b**), resistant *C. auris* compared with normal *C. auris* (**c**), resistant *C. albicans* compared with *P. kudriavzevii* (**d**), normal *C. albicans* compared with *P. kudriavzevii* (**e**), resistant *C. albicans* compared with resistant *N. glabrata* (**f**), and normal *C. albicans* compared with resistant *N. glabrata* (**g**).

The protein-protein interaction network among DEMPs from each pair of strains was analyzed using the STRING database to identify the key proteins associated with azole resistance in each species. As shown in Fig. 5a, azoles resistance in *C. albicans* had a profound impact on mitochondrial-associated cellular processes, resulting in the clustering of only 42 mitochondrial proteins (10%) into three main groups, with UTP20, RSC58, and HGT6 as the centers, respectively. In the *C. auris* set, only 19 mitochondrial proteins formed a visual network, with a prominent junction at the RPS1-rRNA process, which was linked to GPM1 involved in glycolysis, cytochrome-*c* oxidase COX12, mitochondrial aldehyde dehydrogenase ALD5, glutathione peroxidase, heat-shock protein SSA2, and eisosome protein LSP1 (Fig. 5b). Equally, Fig. 5c illustrated a significant interconnectivity among 40 DEMPs associated with the rRNA process in *N. glabrata*.

In parallel with Fig. 5c, ACT1/TUB1-associated cytoskeleton formation, ACC1 hub, and mitochondrial OXPHOS were also clearly showed in Fig. 5d and e when resistant *N. glabrata* (Y10b) compared with susceptible (C103) or resistant *C. albicans* (1052) strains. Particularly, aa synthesis and mitochondrial tRNA synthases only showed in Fig. 5d and e. As shown in Fig. 5f (*P. kudriavzevii* vs susceptible *C. albicans*) and Fig. 5g (*P. kudriavzevii* vs resistant *C. albicans*), two network diagrams were formed by the interaction of 123 and 104 mitochondrial proteins, respectively, and clustered in similar patterns as shown in Fig. 5c and Fig. 5d. However, the rRNA process clusters in *P. kudriavzevii* are less compacted along with more expansion on proteins related to carbon metabolism and mitochondrial TCA and OXPHOS, particularly complex V subunits. Such changes are more distinct when compared with susceptible *C. albicans* (Fig. 5g).

**Fig 6:**
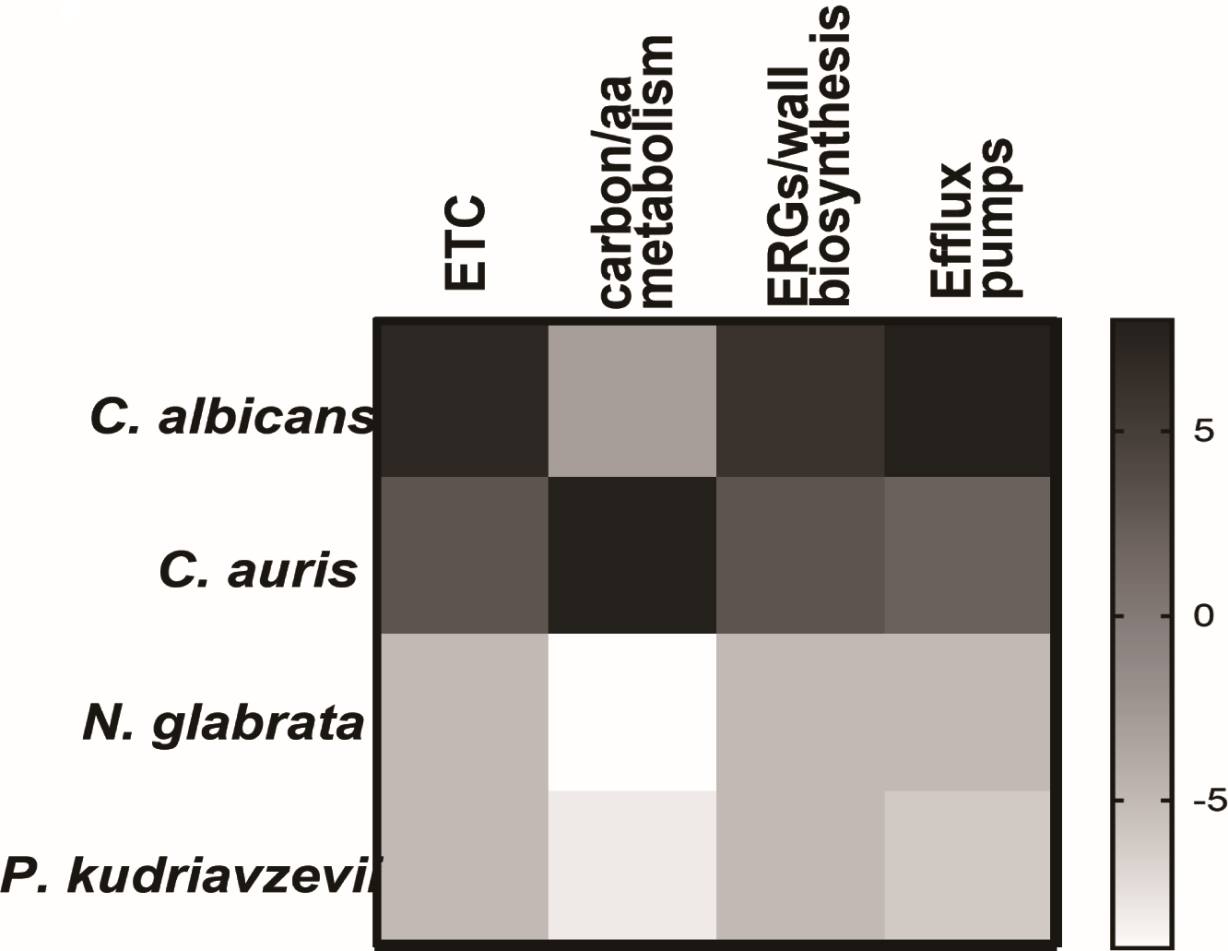
A heat map of the effects of common PPI on *Candida* species. Differences and similarities in mitochondrial mechanisms mediating azole resistance in different *Candida* species.

Taken together, resistance to azoles in each species resulted in common effects on rRNA biogenesis, nuclear pore complex, actin-mitochondrial dynamics, and carbon metabolism. Nevertheless, the degree of effects varied with species (Fig. 6).

### Altered mitochondrial respiration and carbon metabolism were observed in different *Candida* species

Both DEMPs in *N. glabrata* (Y10b vs Y104) and DEMPs in *P. kudriavzevii* (C103 or 1052 vs C6a) were mostly downregulated (Fig. 2b, d and e). Furthermore, several subunits of OXPHOS complex V (CV) and CIV proteins (COX5, COX6) were less translated. The downregulation of IDH2 (isocitrate dehydrogenase 2), PYC2 (pyruvate carboxylase 2), and ACC1 (acetyl-CoA carboxylase) may be related to the inhibition of Snf1 (sucrose non-fermenting 1) in *N. glabrata* (Fig. 5c). The reduction HMG-CoA reductase may decelerate sterol biosynthesis and ribosomal biogenesis in resistant *N. glabrata* and *P. kudriavzevii*. However, three key enzymes for glycolysis and gluconeogenesis, namely, alcohol dehydrogenase 1, enolase, and 3-phosphoglycerate kinase, were only increased in *P. kudriavzevii* (Fig. 2d).

Conversely, DEMPs in *C. auris* (12768 vs 10913) were mostly upregulated (Fig. 2c). Among these DEMPs, the proteins related to carbon metabolism (GPM1, ALD5, ADH2) and mitochondrial respiration (COX2, COX12) were upregulated, while TOM40 was downregulated. Among DEMPs in *C. albicans* (1052 vs C103), proteins for OXPHOS (COX5, PET17, ATP2), AOX2, TOM6, TIM12, and SAM37 were markedly upregulated (Fig. 2a). In addition, three proteins (NAD3, NUDFB10, and orf19. 4324) for CI, cytochrome *c* protein (CYT1), ubiquinone synthase COQ5, and a few mitochondrial ribosome-associated proteins were significantly increased in resistant *C. albicans* only (Fig. 2a). However, proteins related to carbon metabolism were inhibited, including ACO1, ARA2, SPE3, MAE1, and PTH1 (Fig. 2a).

## DISCUSSION

Both *C. albicans* and *C. auris* belong to the CTG clade (24). Here, our results showed that *C. albicans* and *C. auris* shared similar effects on mitochondrial respiration and ergosterol and cell wall polysaccharide synthesis under azole stress (Fig. 6). In addition, *P. kudriavzevii* and *N. glabrata* that did not belong to the CTG clade performed similarly in low levels of TCA enzymes, amino acid synthases, ERGs, and mannan transferases. This indicated that different *Candida* species in the same category seemed to have many commonalities in mediating the mitochondrial mechanism of azole resistance.

*N. glabrata* is much more closely related to fermentable *Saccharomyces cerevisiae* than to *C. albicans*, especially in aspects of its energy metabolism (25). Like *S. cerevisiae, N. glabrata* has no CI in OXPHOS and is a Crabtree positive organism that can rapidly convert glucose to ethanol and carbon dioxide under both anaerobic and aerobic conditions. *C. krusei* is already being considered the anamorphic form of *P. kudriavzevii*, which is renowned for its fermentation capabilities and potential as a bioethanol producer. At the proteomic level, the entire suppression of mitochondrion-associated respiration (via CIV and CV), TCA enzymes, hexose transporters, enzymes for glycolysis, and ERGs for ergosterol in resistant *N. glabrata* was identical to the suppression found in *P. kudriavzevii*.

Furthermore, magnificently downregulated DEMPs persist in resistant *C. glabrata* or *P. kudriavzevii* even though we switched the strain used for comparison to resistant *C. albicans*. Since the downregulation of these energy metabolic processes is evident in resistant *N. glabrata* vs its own susceptible strain, such suppression with both resistant and susceptible *C. albicans* suggests the downregulation of mitochondrion-associated activities arisen in *N. glabrata and P. kudriavzevii* are unrelated to the metabolic responses in resistant *C. albicans*. Nevertheless, the drug resistance is uniformly associated with stepdown energy metabolism in these two fermenter species.

Our data reveal that the responsible regulators for energetic metabolism under azole resistance are different between *C. albicans* and two fermenter species. The heat shock proteins (Hsp60, Hsp70, and Hsp90) increased in CTG species but decreased in non-CTG fermenter species. In addition, in any *N. glabrata* or *P. kudriavzevii* scenario, Snf1 complex, Tor1 kinase, and/or MAP kinase Hog1 were severely depressed, which certainly is the basis of the step-down expression of ribosomal biogenesis, cell cyclins, cytoskeleton activities, and carbon metabolism, which are all classical down-stream cellular activities regulated by them. In yeasts, an integrated TORC1 and PKA signaling have been associated with the temporal activation of glucose-induced gene expression (26). Also, the Snf1 and the Hog1 MAPK regulate global changes in gene expressions for utilizing alternate carbon sources in Crabtree-positive yeasts upon phosphorylation (27, 28), which perhaps explains the downregulation of mitochondrial OXPHOS CIV and CV in Crabtree-positive *N. glabrata* and *P. kudriavzevii* in this study due to suppressive response of these energic regulators. Apparently, the inactivation of these metabolic signaling pathways promotes survival during resistance evolution in both species (22).

In contrast, the activation responses in ribosomal biogenesis, ergosterol synthesis, cell wall polysaccharide synthesis, and efflux pumps in resistant *C. albicans* and *C. auris* produce no changes in the content of Tor1, Snf1, and Hog1. However, Tor1-activator protein kinase (Ksp1) and phosphohistidine intermediate protein (Ypd1) in the Hog1 pathway were over-produced. According to Chang et al., Ksp1 in *S. cerevisiae* combines the Snf1/AMPK and TORC1 signaling pathways by increasing autophagy and regulating post-transcriptional events during glucose deprivation (29). Since these universal energic regulators are expected to act differently in *C. albicans*, the increased but still dampened DEMPs on ribosomal biogenesis could be an artifact of the combined highly translated Ksp1 and Ypd1 or by Ypd1 alone. Ypd1 is the upstream intermediate protein in the Hog1 MAPK signaling, which activates phosphorylation of Ssk1, a repressor for Hog1 activation. Therefore, the increased Ypd1 in *C. albicans* eventually suppresses the Hog1 response, a similar consequence to what was noted in *N. glabrata*. The similar downregulated Hog1 response can explain the slightly decreased response on OXPHOS CIV-CV activity and glycolysis in *C. albicans* as well. However, we need to find a better explanation for the unforeseen activation of CI activity of OXPHOS found only in resistant *C. albicans*. These data highlight the connection between azole treatment and suppression of the classical respiration process, potentially acting through the sequence of CIII, CV, and CI.

With the exception of a few protein kinases, phophotase Ptc7 and Reg1, six transcription factors were found to be increased in resistant *C. albicans*, showing high activity in the biosynthesis of ergosterol, mannan, and glucan on cell walls. Among these transcription factors, Zcf29 and Spt7 have no functional annotations; Msn4 and Cap4 are general stress regulators, and Sef1 is required for iron uptake. The last transcription factor—Rtg3 (3.8-fold increased) has been better characterized than the other 5 transcription factors in regulating energic metabolism. In eukaryotic cells, Rtg3, working with Rtg1, acts as a retrograde regulator to initiate the transcription of a group of genes for galactose catabolism in response to dysfunctional mitochondrial respiration (30). The outcome of this retrograde pathway is profound readjustments of carbohydrate and nitrogen metabolism. In fungi, this system is mostly studied in *S. cerevisiae* and is less well-studied as a factor in any energy crisis in *C. albicans*.

Although we do not know the direct link between azole stress and retrograde regulation pathways, changes in intracellular Ca^2+^ dynamics related to mitochondria is the initiating signal for retrograde responses in mammalian cells (31, 32). At least, the calcium signaling is required for survival of azole stress in *N. glabrata* (32). Nevertheless, the replenishment of glutamate and acetyl CoA in TCA and alternative nitrogen uptake are the signatures of a “successful” compensation for mitochondrial inefficiency via retrograde regulation (33), which seem to be reflected in resistant *C. albicans* here. We observe activation of peroxins (Pex2, Pex6, Pex22, and orf19.2168.3 for peroxisome assembly and β-oxidation) and Fnx1 (the drug:proton antiporter) and increased proteins for nitrogen and NH_3_^+^ utilization including Ato2, Frp3, Mho1. Meanwhile, the decrease of glutamate decarboxylase (GAD1) will decelerate the breakdown of glutamate within mitochondria. According to Butow and Avadhani, the reason for replenishment of glutamate is due to the blockage from succinate to fumarate, oxaloacetate, and, in turn, to ɑ-ketoglutarate in TCA, the latter being a direct precursor of glutamate. Indeed, the enzymes involved in glutamate or TCA intermediates in mitochondria are decreased in resistant *C. albicans*, which include Aco1 for glyoxylate cycle, malate decarboxylase Mae1 for converting malate to pyruvate, and Bpl1 for acetyl CoA carboxylase and pyruvate carboxylase. Although none of the CII subunits were changed in resistant *C. albicans*, the low flux of TCA intermediates or glutamate promotes the retrograde response. We believe that a robust retrograde response is the key player in coordinating the metabolic flow (biogenesis and metabolism) during azole resistance evolution in *C. albicans*, which ensure the sufficient energy supply for competing with other fermenter organisms.

The BN-PAGE shows that the CI assembly seems more tolerant than CV and CIII to fluconazole in *C. albicans* in this study, which could be explained by a competent Rtg3 response in *C. albicans*. We note that the decreased CIV/CV response is more universal in every *Candida* spp. tested here. The increased CI activity in resistant *C. albicans* is also supported by increases of an anti-mitochondrial fission protein (orf19.2961), TIMs and TOMs. Together with more tolerance of CI under fluconazole, it highlights that an upregulated CI activity is a part of retrograde response that was induced by azole stress in *C. albicans*. Obviously, when the classified downstream targets of retrograde response were proposed in *S. cerevisiae*, which lacks CI, the activation route via signals from azole, Ca_2_^+^ dynamics, to retrograde response and downstream mitochondria OXPHOS activity, need to be clarified in *C. albicans*.

In summary, these data suggested that the expression of mitochondrial proteins was highly likely to be associated with azoles resistance in fungi. Activation of the retrograde responses in *C. albicans* and the decreases of TORC, Snf complex and the MAPK Hog1 pathways in fermenter *Candida* spp. secure their growth during the azole resistance. When the Tor1 or the Hap complex can be used to interpret the more universal decreases of TCA and CIV/CV-mediated respiration in mitochondria, the upregulation of CI activity is likely a part of retrograde response triggered by azole stress in *C. albicans*.

## Data Availability

The mass spectrometry proteomics data have been deposited in the ProteomeXchange Consortium (http://proteomecentral.proteomexchange.org) via the PRIDE partner repository with the data set identifier PXD045101.
